# Supplementary material for: Comprehensive Simulation of Ca2+ Transients in the Continuum of Mouse Skeletal Muscle Fiber Types
Source: Int J Mol Sci. 2021 Nov 17;22(22):12378. doi: 10.3390/ijms222212378 (PMC8624975; doi:10.3390/ijms222212378)
Supplement: Supplementary file 1 [file ijms-22-12378-s001.zip › ijms-1391248-Supplementary.pdf]

International Journal of
Molecular Sciences

MDPI

Supplementary Material

Comprehensive Simulation of Ca^{2+} Transients in the Continuum of Mouse Skeletal Muscle Fiber Types

Oscar A. Rincón [1,2], Andrés F. Milán [2] Juan C. Calderón [2] and Marco A. Giraldo [1,*]

[1] Biophysics Group, Institute of Physics, University of Antioquia, Medellín, Colombia.
[2] Physiology and Biochemistry research Group-PHYSIS, Faculty of Medicine, University of Antioquia, Medellín, Colombia
* Correspondence: mantonio.giraldo@udea.edu.co; Tel.: +573015440055

The simulation of tetanic Ca^{2+} transients was extended during 10 s and the mechanisms that required more time to return to equilibrium are presented in Fig S1. In the sarcoplasm, PV and TNS returned to their resting states after 6 ms. About the same time was required for CSQ in the SR. The mitochondrial buffers require more than 10 ms. The transmembrane fluxes also remained activated for time intervals greater than 6 s. In fibers IIX and IIB, the fluxes maintained their rate for a longer time, presumably due to the Ca^{2+} stored in the sarcomplasmic binding sites and in the SR.

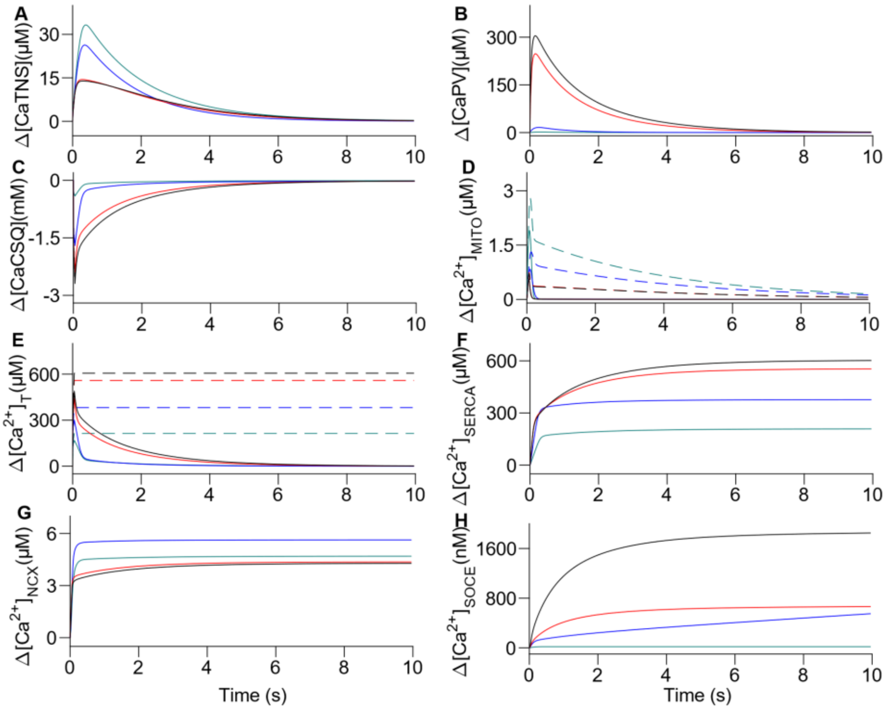

Figure S1. Simulation of tetanic Ca^{2+} transients during an extended time interval. The $\Delta[Ca^{2+}]$ coupled to the high affinity sarcoplasmic buffers: TNS (**A**) and PV (**B**); the SR buffer: CSQ (**C**); the free (solid line) and total $\Delta[Ca2+]_{MITO}$ (dashed line) (**D**) are shown. The total $[Ca^{2+}]$ in the sarcoplasm (solid line) and the total $[Ca^{2+}]$ released (dashed line) were obtained following the same procedure as in Fig. 2I (**E**). The integral of the fluxes in time across the sarcolemma, produced by the SERCA pump, the NCX and the SOCE, were also obtained (**F**, **G**, **H**).

Table S1. Rate constants used in the model for Ca^{2+} and Mg^{2+} binding reactions at 22°C.

Binding sites	k_+	k_-	Reference
Ca^{2+}-Tn	2.68×10^8 M^{-1} s^{-1}	2340.2 s^{-1}	[16] & Present work
Ca^{2+}-CaTn	1.34×10^8 M^{-1} s^{-1}	25.9 s^{-1}	[16] & Present work
Ca^{2+}-TNS	2.44 M^{-1} s^{-1}	0.488 s^{-1}	[13] & Present work
Mg^{2+}-TNS	8.12 M^{-1} s^{-1}	1.62 s^{-1}	[13] & Present work
Ca^{2+}-PV	0.632×10^8 M^{-1} s^{-1}	0.8 s^{-1}	[16] & Present work
Mg^{2+}-PV	0.5×10^5 M^{-1} s^{-1}	4.5 s^{-1}	[16] & Present work
Ca^{2+}-ATP	0.2067×10^8 M^{-1} s^{-1}	45.5 s^{-1}	[16] & Present work
Ca^{2+}-CSQ	44×10^6 M^{-1} s^{-1}	3446 s^{-1}	[27] & Present work
Ca^{2+}-B	0.6×10^6 M^{-1} s^{-1}	0.156 s^{-1}	[14] & Present work

References

16. Baylor, S.M.; Hollingworth, S. Simulation of Ca2+ Movements within the Sarcomere of Fast-Twitch Mouse Fibers Stimulated by Action Potentials. J. Gen. Physiol. 2007, 130, 283–302, doi:10.1085/jgp.200709827.

13. Hollingworth, S.; Kim, M.M.; Baylor, S.M. Measurement and simulation of myoplasmic calcium transients in mouse slow-twitch muscle fibres. J. Physiol. 2012, 590, 575–594, doi:10.1113/jphysiol.2011.220780.

27. Barclay, C.J.; Launikonis, B.S. Components of activation heat in skeletal muscle. J. Muscle Res. Cell Motil. 2021, 42, doi:10.1007/s10974-019-09547-5.

14. Marcucci, L.; Canato, M.; Protasi, F.; Stienen, G.J.M.; Reggiani, C. A 3D diffusional-compartmental model of the calcium dynamics in cytosol, sarcoplasmic reticulum and mitochondria of murine skeletal muscle fibers. PLoS One 2018, 13, e0201050, doi:10.1371/journal.pone.0201050.
